# Predictors of lack of glycemic control in persons with type 2 diabetes

**DOI:** 10.1186/s40842-023-00160-7

**Published:** 2024-01-25

**Authors:** Judy Z. Louie, Dov Shiffman, Charles M. Rowland, Norma S. Kenyon, Ernesto Bernal-Mizrachi, Michael J. McPhaul, Rajesh Garg

**Affiliations:** 1 Quest Diagnostics Nichols Institute, 33608 Ortega Highway, San Juan Capistrano, CA 92675 USA; 2 Diabetes Research Institute, Miller School of Medicine, 1951 NW 7Th Avenue, Miami, FL 33136 USA; 3 Comprehensive Diabetes Center, Division of Endocrinology, Diabetes, and Metabolism, 5555 Pone de Leon Blvd, Coral Gables, FL 33136 USA; 4 Present address: The Lundquist Research Institute at Harbor-UCLA, Liu Research Building, Room 212, 1124 W. Carson Street, Torrance, CA 90502 USA

**Keywords:** Type 2 diabetes, Glycemic Control, Health disparities, Neighborhood, Health Insurance, Socioeconomic

## Abstract

**Background:**

Professional guidelines recommend an HbA1c < 7% for most people with diabetes and < 8.5% for those with relaxed glycemic goals. However, many people with type 2 diabetes mellitus (T2DM) are unable to achieve the desired HbA1c goal. This study evaluated factors associated with lack of improvement in HbA1c over 3 years.

**Methods:**

All patients with T2DM treated within a major academic healthcare system during 2015–2020, who had at least one HbA1c value > 8.5% within 3 years from their last HbA1c were included in analysis. Patients were grouped as improved glycemic control (last HbA1c ≤ 8.5%) or lack of improvement (last HbA1c > 8.5%). Multivariate logistic regression analysis was performed to assess independent predictors of lack of improvement in glycemic control.

**Results:**

Out of 2,232 patients who met the inclusion criteria, 1,383 had an improvement in HbA1c while 849 did not. In the fully adjusted model, independent predictors of lack of improvement included: younger age (odds ratio, 0.89 per 1-SD [12 years]; 95% CI, 0.79–1.00), female gender (1.30, 1.08–1.56), presence of hypertension (1.29, 1.08–1.55), belonging to Black race (1.32, 1.04–1.68, White as reference), living in low income area (1.86,1.28–2.68, high income area as reference), and insurance coverage other than Medicare (1.32, 1.05–1.66). Presence of current smoking was associated with a paradoxical improvement in HbA1c (0.69, 0.47—0.99). In a subgroup analysis, comparing those with all subsequent HbA1c values > 8.5% (*N* = 444) to those with all subsequent HbA1c values < 8.5% (*N* = 341), similar factors were associated with lack of improvement, but smoking was no longer significant.

**Conclusion:**

We conclude that socioeconomic factors like race, type of insurance coverage and living in low-income areas are associated with lack of improvement in HbA1c over a period of 3-years in people with T2DM. Intervention strategies focused on low-income neighborhoods need to be designed to improve diabetes management.

**Supplementary Information:**

The online version contains supplementary material available at 10.1186/s40842-023-00160-7.

Type 2 diabetes mellitus (T2DM) is one of the most common chronic diseases, affecting millions of Americans [1]. Poorly controlled T2DM is associated with chronic complications that can have devastating consequences for the patient. Randomized controlled trials have shown that good glycemic control can prevent the chronic complications of diabetes [2]. The American Diabetes Association (ADA) recommends an HbA1c < 7% for most people with diabetes and higher HbA1c for those with relaxed glycemic goals due to the presence of comorbidities or other considerations [3]. However, despite major advancements in the management of diabetes, including the availability of several new classes of anti-diabetic drugs, a large proportion of people with T2DM are unable to achieve the desired HbA1c goals [4, 5]. While it has been possible to achieve tight glycemic control in clinical trial settings [6, 7], achieving tight glycemic control in real-life clinical practice has been difficult. The reasons for inability to achieve HbA1c goals in many people with T2DM in the clinical setting have not been fully explored.

Several psychosocioeconomic factors along with medical factors can affect a patient’s ability to achieve adequate glycemic control. A better understanding of these factors may help in designing better treatment strtegies. Most of the previous studies on the association between socioeconomic factors and HbA1c were crossectional and provided limited information [8, 9]. We conducted a longitudinal data analysis to understand factors associated with lack of glycemic control in patients receiving clinical care within a major academic healthcare system. This study was performed to evaluate the predictors of lack of improvement in HbA1c over a period of 3 years. Another goal of this study was to assess the geographical location of most-at-need populations in preparation for interventions to improve clinical care.

## Methods

This was a retrospective study including patients with T2DM managed at a major academic center. Waiver of informed consent was approved by the Institutional Review Board. The study was performed in accordance with the ethical standards of the 1964 Helsinki declaration and its later amendments. It was not possible to involve patients or the public in the design, conduct, or reporting of this research. All patients aged > 18 years with T2DM based on ICD-10 code (E11) on two occasions and who were treated at one of the health system’s clinics between Jan 2015 and Dec 2020 were included in data analysis. Our healthcare system's comprehensive network includes three hospitals and more than 30 outpatient facilities. Patients with ICD-10 codes for other than T2DM (E08, E09, E10, E12 or E13) at any time in the database were excluded.

Patient demographic data and laboratory data were obtained from electronic medical records in early 2021. The latest available values for all variables were included in the data analysis. Hypertension was defined as systolic blood pressure ≥ 140 mmHg or diastolic blood pressure ≥ 90 mmHg. BMI was classified into 3 categories < 25, 25 to < 30, and ≥ 30 kg/m^2^. Income area was based on residence ZIP codes and classified as low income for ZIP codes with < 60% of state median, low-medium for ZIP codes with 60 to 100% of state median, high-medium for ZIP codes with > 100 to 140% of state median and high for ZIP codes with > 140% of state median based on the 2019 American Community Survey conducted by the U.S. Census Bureau [10]. Marital status was classified as married status including those with a domestic partner and single status including those that were divorced, widowed, or legally separated. Last visit year was based on the last encounter with a primary care physician or an endocrinologist.

### Statistical analysis

Patients were included in the final analysis if they had at least one HbA1c value available in each of 3 years prior to current HbA1c and ≥ 1 of those values was > 8.5% (Supplementary table 1). Patients with improved glycemic control (last HbA1c ≤ 8.5%) were compared with those with lack of improvement (last HbA1c > 8.5%).

The continuous variables were reported as medians and interquartile ranges, and the categorical variables were reported as counts and percentages. Differences in patient characteristics between those with improved glycemic control and those with lack of improvement were assessed with Wilcoxon rank sum test for continuous variables and Chi-square test for categorical variables. The association of each variable with lack of improvement in glycemic control was assessed with a logistic regression model that was adjusted for age and gender. The effect of age was evaluated per 1 standard deviation (SD). The independent association of each variable with lack of improvement was assessed in a logistic regression model that included all variables as well as the service facility. In addition, a risk score for all patients was estimated in a logistic regression that included risk factors significantly predicting lack of improvement. Two-sided *p* value < 0.05 was considered significant. All analyses were performed in R software.

## Results

**Fig. 1:**
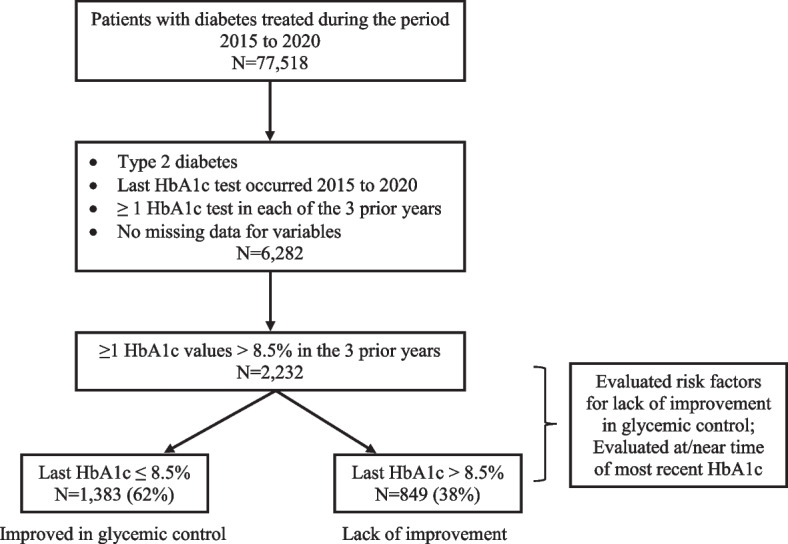
Study population flow diagram

Patient inclusion for data analysis is shown in Fig. 1. The study included a total of 2,232 patients with median (interquartile) age of 63 (57 -70) years. Almost half (49.8%) of the patients were women. Most of the patients (67.7%) were self-reported White and 23.9% were self-reported Black. About 62% patients had improvement in glycemic control (last HbA1c ≤ 8.5%) and the rest (38%) had lack of improvement (last HbA1c > 8.5%). The reduction in HbA1c from the maximum value in previous years to the last HbA1c was faster in patients with improvement in glycemic control than those with lack of improvement (the annual median rate of change, -1.0 vs. -0.3; *p* value < 0.0001).

**Fig. 2:**
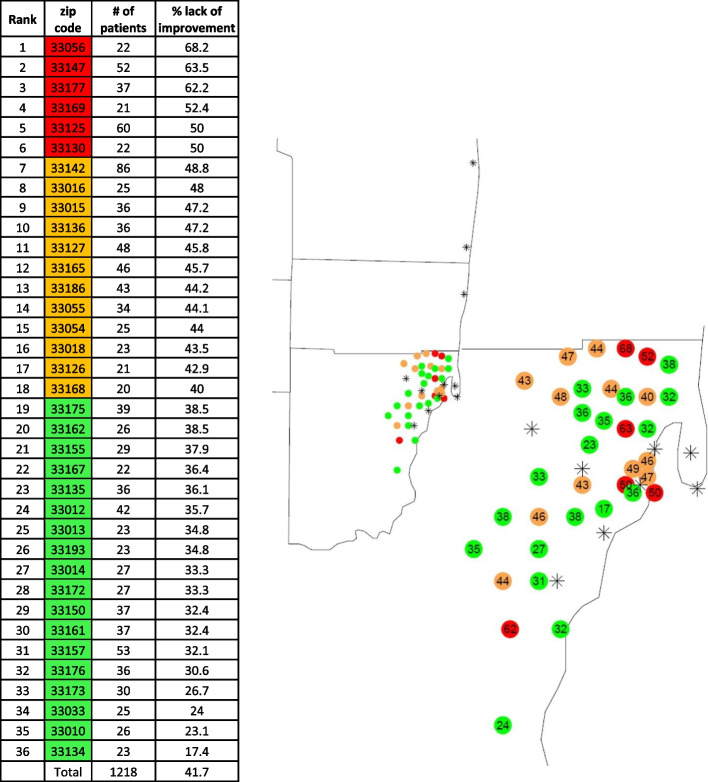
Zip codes with proportion of patients with lack of Improvement. Only the ZIP codes with total number of patients ≥ 20 are shown. The left map panel depicts a high-level view of the Miami area; the right panel shows a more focused area centered on the relevant Zip codes and patient service facilities. In both maps, the approximate locations of the patient service facilities are denoted by the symbol "*"

Comparisons of the variables between patients with improvement in glycemic control and those with lack of improvement are shown in Table 1. Compared to those with improvement, patients with lack of improvement were younger (median age, 62 vs. 64 years; *p* value < 0.0001), more likely to be female (55.5% vs. 46.3%; *p* value < 0.0001), and belong to Black race (28.7% vs. 21.0%; *p* value = 0.0001). Patients with lack of improvement were also more likely to have hypertension (40.9% vs. 34.9%; *p* value = 0.0055), live in low-income area (24.1% vs.18.2%; *p* value = 0.0001), less likely to have Medicare coverage (29.8% vs. 39.5%; *p* value < 0.0001), and less likely to be current smokers (5.7% vs. 8.2%; *p* value = 0.035) than those with improvement. Current smokers had a lower BMI (30 ± 5 kg/m^2^) as compared to all others (32 ± 6 kg/m^2^). Among patients living in six ZIP code areas, ≥ 50% lacked improvement with the highest proportion being 68.2% (Fig. 2). No difference was observed between those with and without improvement for BMI, marital status, preferred language, visit with endocrinology vs. primary care, and last visit year.

**Table 1:** Characteristics according to the status of lack of improvement in glycemic control

	Improved Glycemic Control	Lack of Improvement in Glycemic Control	*P* value
Number of patients, N	1383	849	
Age, year	64 (58—71)	62 (56—68)	< 0.0001
Gender, N (%)			< 0.0001
Female	641 (46.3)	471 (55.5)	
Male	742 (53.7)	378 (44.5)	
Marital status, N (%)			0.17
Married	785 (56.8)	456 (53.7)	
Single	598 (43.2)	393 (46.3)	
Hypertension, N (%)			0.0055
No	900 (65.1)	502 (59.1)	
Yes	483 (34.9)	347 (40.9)	
Race, N (%)			0.0001
Black	290 (21)	244 (28.7)	
White	976 (70.6)	534 (62.9)	
Other	117 (8.5)	71 (8.4)	
BMI, kg/m^2^, N (%)			0.26
< 25	187 (13.5)	97 (11.4)	
25 to < 30	452 (32.7)	271 (31.9)	
≥ 30	744 (53.8)	481 (56.7)	
Income area, N (%)			0.0001
Low	252 (18.2)	205 (24.1)	
Low medium	504 (36.4)	317 (37.3)	
High medium	452 (32.7)	260 (30.6)	
High	175 (12.7)	67 (7.9)	
Preferred Language, N (%)			0.28
English	898 (64.9)	562 (66.2)	
Spanish	462 (33.4)	266 (31.3)	
Other	23 (1.7)	21 (2.5)	
Insurance Coverage, N (%)			< 0.0001
Commercial insurance	732 (52.9)	505 (59.5)	
Medicaid	105 (7.6)	91 (10.7)	
Medicare	546 (39.5)	253 (29.8)	
Smoking, N (%)			0.035
Current	113 (8.2)	48 (5.7)	
Former	440 (31.8)	255 (30.0)	
Never	830 (60.0)	546 (64.3)	
Specialty status, N (%)			0.051
Endocrinology	467 (33.8)	322 (37.9)	
Other	916 (66.2)	527 (62.1)	
Last encounter type, N (%)			0.60
Hospital Encounter	146 (10.6)	80 (9.4)	
Office Visit	987 (71.4)	621 (73.1)	
Telemedicine	250 (18.1)	148 (17.4)	
Last visit year, N (%)			1
2015	66 (4.8)	40 (4.7)	
2016	106 (7.7)	66 (7.8)	
2017	164 (11.9)	100 (11.8)	
2018	197 (14.2)	121 (14.3)	
2019	254 (18.4)	155 (18.3)	
2020	596 (43.1)	367 (43.2)

In the logistic regression model adjusted for age and gender, younger people were more likely to have lack of improvement in glycemic control. The odds of lack of improvement increased by 18% per 1-standard deviation (12 years) decrease in age. Female patients were 43% more likely (OR 1.43; 95% CI 1.20—1.69) to have lack of improvement than males. People with hypertension were 34% more likely to have lack of improvement (OR 1.34; 95% CI 1.12—1.60) than those without hypertension. People belonging to Black race were 39% more likely (OR 1.39; 95% CI 1.13—1.71) to have lack of improvement than people belonging to White race. People in lower income areas were more likely to have lack of improvement. The odds of lack of improvement were almost twofold greater (OR 1.94; 95% CI 1.38—2.73) for low-income area, 56% higher (OR 1.56; 95% CI 1.13—2.14) for low medium income area, 47% higher (OR 1.47; 95% CI 1.07—2.03) for high medium income area than that for high income area. People with commercial and Medicaid insurance coverage were 30% (OR 1.30; 95% CI 1.04—1.63) and 57% (OR 1.57; 95% CI 1.12—2.21) more likely to have lack of improvement, respectively, compared to those with Medicare. The effects of other factors in having lack of improvement were not significant (Table 2).

**Table 2:** Factors associated with lack of improvement in glycemic control after adjusting for age and gender

Factors		OR (95% CI)	*P* value
Age (per 1-SD)		0.82 (0.74—0.89)	< 0.0001
Gender	Female vs. Male	1.43 (1.20 – 1.69)	< 0.0001
Marital status	Single vs. Married	1.04 (0.87—1.24)	0.69
Hypertension	Yes vs. No	1.34 (1.12—1.60)	0.001
Race	Black	1.39 (1.13—1.71)	0.002
	Other	1.07 (0.78—1.46)	0.69
	White (reference)	1.00	
BMI	≥ 30	1.12 (0.85—1.48)	0.42
	25 to < 30	1.13 (0.85—1.51)	0.41
	< 25 (reference)	1.00	
Income area	Low	1.94 (1.38—2.73)	0.0001
	Low medium	1.56 (1.13—2.14)	0.006
	High medium	1.47 (1.07—2.03)	0.019
	High (reference)	1.00	
Preferred language	Spanish	0.96 (0.80—1.16)	0.66
	Other	1.55 (0.85—2.85)	0.16
	English (reference)	1.00	
Insurance coverage	Commercial insurance	1.30 (1.04—1.63)	0.02
	Medicaid	1.57 (1.12—2.21)	0.009
	Medicare (reference)	1.00	
Smoking	Current	0.70 (0.49—1.00)	0.05
	Former	1.00 (0.83—1.22)	0.96
	Never (reference)	1.00	
Specialty status	Endocrinology vs. Other	1.14 (0.95—1.37)	0.15
Last encounter type	Office Visit	1.12 (0.84—1.51)	0.43
	Telemedicine	1.02 (0.72—1.43)	0.93
	Hospital Encounter (reference)	1.00	
Last visit year	2015	1.09 (0.72—1.66)	0.68
	2016	1.07 (0.76—1.50)	0.70
	2017	1.05 (0.79—1.39)	0.75
	2018	1.04 (0.80—1.36)	0.75
	2019	1.02 (0.80—1.30)	0.88
	2020 (reference)	1.00	

In multivariate logistic regression analysis, including all variables and service facility in the model, age, gender, hypertension, race, income area, and insurance coverage remained significant in predicting lack of improvement in glycemic control (Table 3). After accounting for other factors, the effects of the independent predictors of lack of improvement were attenuated slightly for younger age (OR, 0.89 per 1-SD [12 years]; 95% CI 0.79—1.00), female gender (OR 1.30, 95% CI 1.08 – 1.56), presence of hypertension (OR 1.29, 95% CI 1.08—1.55), Black race (OR 1.32, 95% CI 1.04—1.68, vs. White), lower income area (OR 1.86, 95% CI 1.28—2.68, for low income area; OR 1.51, 95% CI 1.09—2.09 for low medium income area; OR 1.47, 95% CI 1.06—2.04 for high medium income area), and insurance coverage other than Medicare (OR 1.32, 95% CI 1.05—1.66, for commercial insurance; OR 1.55, 95% CI 1.09—2.21 for Medicaid). Presence of current smoking was associated with lower chances of lack of improvement in HbA1c (OR 0.69, 95% CI 0.47—0.99) in a model that was adjusted for all other factors.

**Table 3:** Factors associated with lack of improvement in glycemic control after adjusting for all other factors

Factors		OR (95% CI)	*P* value
Age (per 1-SD)		0.89 (0.79—1.00)	0.042
Gender	Female vs. Male	1.30 (1.08 – 1.56)	0.006
Marital status	Single vs. Married	1.00 (0.83—1.21)	0.98
Hypertension	Yes vs. No	1.29 (1.08—1.55)	0.006
Race	Black	1.32 (1.04—1.68)	0.023
	Other	1.05 (0.76—1.45)	0.77
	White (reference)	1.00	
BMI	≥ 30	1.05 (0.79—1.39)	0.74
	25 to < 30	1.10 (0.82—1.48)	0.54
	< 25 (reference)	1.00	
Income area	Low	1.86 (1.28—2.68)	0.001
	Low medium	1.51 (1.09—2.09)	0.014
	High medium	1.47 (1.06—2.04)	0.023
	High (reference)	1.00	
Preferred language	Spanish	0.95 (0.76—1.18)	0.63
	Other	1.40 (0.75—2.62)	0.29
	English (reference)	1.00	
Insurance coverage	Commercial insurance	1.32 (1.05—1.66)	0.017
	Medicaid	1.55 (1.09—2.21)	0.014
	Medicare (reference)		
Smoking	Current	0.69 (0.47—0.99)	0.046
	Former	1.05 (0.86—1.28)	0.65
	Never (reference)	1.00	
Specialty status	Endocrinology vs. Other	1.14 (0.86—1.51)	0.37
Last encounter type	Office Visit	1.24 (0.86—1.78)	0.25
	Telemedicine	1.10 (0.72—1.70)	0.65
	Hospital Encounter (reference)	1.00	
Last visit year	2015	0.98 (0.62—1.53)	0.92
	2016	1.03 (0.71—1.49)	0.89
	2017	0.97 (0.71—1.33)	0.87
	2018	0.98 (0.73—1.32)	0.89
	2019	0.96 (0.73—1.27)	0.79
	2020 (reference)	1.00	

A risk score for predicting lack of improvement for all patients was estimated in a logistic regression model including risk factors: age, gender, race, hypertension, income area, and insurance coverage. The model worked moderately well in predicting lack of improvement in glycemic control. Half of the patients (50%) in the top quartile of the risk score had lack of improvement, compared to 29.2% of those in the bottom quartile. Income area and insurance coverage performed better than race in predicting lack of improvement. For patients in the top quartile of the risk score, 93.2% had commercial insurance and Medicaid, 79.2% were in low medium and low-income areas, 54.7% were of Black race and 59.7% had hypertension, as compared to those in the bottom quartile with 21.2%, 32.1%, 4.5%, and 20.9% for the same parameters respectively.

In those with the lack of improvement, 444 (52.3%) patients had consistent lack of improvement with all four-year HbA1c values > 8.5% (Group 1 in Supplementary table 1). In those with improved glycemic control, 341 (24.7%) patients had consistent improvement with one initial year HbA1c > 8.5% and all subsequent year HbA1c values ≤ 8.5% (Group 11 in Supplementary table 1). We conducted a subgroup analysis of comparing the distributions of all factors between these two groups. This analysis (supplementary table 2) showed similar results with stronger statistical associations except for smoking that was no longer associated with improvement in glycemic control.

## Discussion

This study shows that lack of improvement in HbA1c levels is common (38%) in people with T2DM receiving treatment at a major academic health center. Socioeconomic factors were associated with persistently high HbA1c, and to a large degree explained the inability to achieve a lower HbA1c over time. Patient related factors, including younger age, female gender and minority racial group, as well as disease related factors such as hypertension, were also associated with lack of improvement in glycemic control; however, socioeconomic factors such as type of insurance coverage, income and neighborhood were the strongest predictors of lack of improvement in HbA1c. These findings provide clues as to possible interventions to improve glycemic control in people with T2DM.

Previous studies have shown that only about 50% people with T2DM achieve an HbA1c < 7%, and about 63% achieve an individualized goal HbA1c [4]. These numbers have remained unchanged over the last two decades, despite pharmaceutical and technological advances in the field [4, 5]. This observation suggests that the medical community may have been missing critical aspects of diabetes management, or that the management of these factors is difficult and currently beyond the reach of medical care providers. Identification of these factors may lead to development of more successful strategies to improve diabetes care. One previous study that used retrospective data and modeling methods showed that most of the factors affecting improvement in HbA1c are, in-fact, nonmodifiable by the clinician [11].

Our study adds to existing data that showed an association between socioeconomic factors and HbA1c in people with diabetes [8, 12]. However, most of the other studies were cross-sectional. One previous longitudinal study over 1 year showed that lack of improvement in HbA1c was associated with younger age, non-white race, Medicaid and other non-Medicare/private health insurance [11]. Our study is unique because it looked at 3 years of longitudinal data and evaluated change in HbA1c over time. Our population was also unique in that all the patients were treated at an academic healthcare center and were highly likely to have received optimal medical interventions as they returned to clinic for repeated assessments. Therefore, these data are widely applicable, even in countries with universal healthcare coverage. A previous cross-sectional study showed higher HbA1c in younger people with T2DM and suggested lack of awareness or access to care [13]. Previous data also show a smaller decrease in HbA1c in women as compared to men on starting insulin treatment [14]. Thus, our findings that younger age, female gender and presence of hypertension were independently associated with lower chances of improvement in HbA1c over time are consistent with these other observations. However, contrary to general belief, we found an association between current smoking and improvement in HbA1c. While cross-sectional studies have shown an association between smoking and higher glucose levels in T2DM [15], there are no prospective data on the association of smoking with glycemic control. Patients with current smoking often have a lower BMI, and this was true in our study as well. However, association of smoking with improved glycemic control was still present after controlling for BMI and needs further investigation.

We think the most important findings in our study were the association between insurance coverage and income area and the chances of improvement in glycemic control. These factors, though independently associated with lack of improvement in our study, are linked to one another. We were able to identify ZIP codes with the highest proportion of patients with inadequate response. These ZIP codes were also inhabited by a higher proportion of Medicaid recipients and people with low income. Previous studies have shown an association between neighborhood and glycemic control [9, 12]. Previous data have also shown an association between type of insurance coverage and quality of care [16]. While lack of insurance would be expected to be associated with inadequate clinical care, expansion of Medicaid coverage was not associated with much of a change in diabetes related outcomes [17]. Thus, neighborhood may be a more important factor in receiving adequate diabetes care. There is a difference in adherence and healthcare utilization depending on the residential ZIP codes of patients [8, 18]. We think there is an opportunity to improve access to diabetes care and improve glycemic control in low-income areas despite many factors being beyond the control of medical professionals.

Our study has limitations in addition to those expected due to its retrospective study design. Ideally, good glycemic control should be defined on the basis of individualized HbA1c goals. We defined poor control as HbA1c > 8.5% in this study because any patient above this level is unlikely to be considered to have good glycemic control. This led to inclusion of many patients with higher than their target HbA1c (< 7% in majority of patients) in the good control category. Thus, the study is biased towards identifying factors associated with more extreme hyperglycemia. We did not have data on the interventions or treatment changes between baseline and final HbA1c, and therefore, we cannot comment on the effectiveness of treatment strategies. We did not have data on the duration of diabetes that could affect response to treatment. Moreover, we included variables at the last visit in data analysis and any changes between initial HbA1c and last HbA1c value may have been missed in this analysis. Finally, we did not have data on variables that can affect adherence to treatment, e.g., psychological health, substance abuse, educational status, and family support.

In conclusion, our results demonstrate that socioeconomic factors are a strong predictor of lack of improvement in HbA1c and that the most affected population lives in certain geographic areas. We were able to identify ZIP codes with the highest proportion of patients with lack of improvement, providing an opportunity to design local interventions in those neighborhoods. It is likely that interventions in the communities rather than in the medical care facilities will be more effective. Further studies will determine the specific interventions that may be successful in these areas.

### Supplementary Information

**Additional file 1:** **Supplementary table 1.** HbA1c status in four studied years.

** Additional file 2:** **Supplementary table 2.** Characteristics according to the status of consistent lack of improvement in glycemic control.

## Data Availability

The data that support the findings of this study are not openly available due to reasons of sensitivity and are available from the corresponding author upon reasonable request.
