# Supplementary material for: Predictors of lack of glycemic control in persons with type 2 diabetes
Source: Clin Diabetes Endocrinol. 2024 Jan 25;10:2. doi: 10.1186/s40842-023-00160-7 (PMC10809600; doi:10.1186/s40842-023-00160-7)
Supplement: Supplementary file 1 — Additional file 1: Supplementary table 1. HbA1c status in four studied years. [file 40842_2023_160_MOESM1_ESM.docx]

Supplementary table 1. HbA1c status in four studied years

|  |  | Prior year HbA1c^a^ | | |  |  |  |
| --- | --- | --- | --- | --- | --- | --- | --- |
| Glycemic Control Status | Group | 3 | 2 | 1 | Last HbA1c | Number of Patients | Fraction (%) |
| Lack of improvement (N=849 patients) | **1** | **>8.5** | **>8.5** | **>8.5** | **>8.5** | 444 | 52.3 |
|  | 2 | **>8.5** | **>8.5** | ≤8.5 | **>8.5** | 66 | 7.8 |
|  | 3 | **>8.5** | ≤8.5 | **>8.5** | **>8.5** | 68 | 8.0 |
|  | 4 | **>8.5** | ≤8.5 | ≤8.5 | **>8.5** | 58 | 6.8 |
|  | 5 | ≤8.5 | **>8.5** | **>8.5** | **>8.5** | 85 | 10.0 |
|  | 6 | ≤8.5 | **>8.5** | ≤8.5 | **>8.5** | 34 | 4.0 |
|  | 7 | ≤8.5 | ≤8.5 | **>8.5** | **>8.5** | 94 | 11.1 |
| Improved (N=1,383 patients) | 8 | **>8.5** | **>8.5** | **>8.5** | ≤8.5 | 273 | 19.7 |
|  | 9 | **>8.5** | **>8.5** | ≤8.5 | ≤8.5 | 154 | 11.1 |
|  | 10 | **>8.5** | ≤8.5 | **>8.5** | ≤8.5 | 103 | 7.4 |
|  | **11** | **>8.5** | ≤8.5 | ≤8.5 | ≤8.5 | 341 | 24.7 |
|  | 12 | ≤8.5 | **>8.5** | **>8.5** | ≤8.5 | 119 | 8.6 |
|  | 13 | ≤8.5 | **>8.5** | ≤8.5 | ≤8.5 | 173 | 12.5 |
|  | 14 | ≤8.5 | ≤8.5 | **>8.5** | ≤8.5 | 220 | 15.9 |

^a^ At least one HbA1c value > 8.5% in a prior year
