# Supplementary material for: Predictors of lack of glycemic control in persons with type 2 diabetes
Source: Clin Diabetes Endocrinol. 2024 Jan 25;10:2. doi: 10.1186/s40842-023-00160-7 (PMC10809600; doi:10.1186/s40842-023-00160-7)
Supplement: Supplementary file 2 — Additional file 2: Supplementary table 2. Characteristics according to the status of consistent lack of improvement in glycemic control. [file 40842_2023_160_MOESM2_ESM.docx]

Supplementary table 2

Characteristics according to the status of consistent lack of improvement in glycemic control.

|  | Consistent Improved Glycemic Control^a^ | Consistent Lack of Improvement in Glycemic Control^b^ | P value |
| --- | --- | --- | --- |
| Number of patients, N | 341 | 444 |  |
| Age, year | 64 (57 - 71) | 61 (56 - 67) | 0.0004 |
| Gender, N (%) |  |  | <0.0001 |
| Female | 138 (40.5) | 264 (59.5) |  |
| Male | 203 (59.5) | 180 (40.5) |  |
| Marital status, N (%) |  |  | 0.0021 |
| Married | 212 (62.2) | 226 (50.9) |  |
| Single | 129 (37.8) | 218 (49.1) |  |
| Hypertension, N (%) |  |  | 0.0014 |
| No | 231 (67.7) | 250 (56.3) |  |
| Yes | 110 (32.3) | 194 (43.7) |  |
| Race, N (%) |  |  | 0.0024 |
| Black | 77 (22.6) | 150 (33.8) |  |
| White | 238 (69.8) | 261 (58.8) |  |
| Other | 26 (7.6) | 33 (7.4) |  |
| BMI, kg/m^2^, N (%) |  |  | 0.50 |
| <25 | 47 (13.8) | 49 (11) |  |
| 25 to <30 | 106 (31.1) | 140 (31.5) |  |
| ≥30 | 188 (55.1) | 255 (57.4) |  |
| Income area, N (%) |  |  | <0.0001 |
| Low | 49 (14.4) | 128 (28.8) |  |
| Low medium | 134 (39.3) | 172 (38.7) |  |
| High medium | 107 (31.4) | 113 (25.5) |  |
| High | 51 (15.0) | 31 (7.0) |  |
| Preferred Language, N (%) |  |  | 0.32 |
| English | 232 (68.0) | 280 (63.1) |  |
| Spanish | 101 (29.6) | 154 (34.7) |  |
| Other | 8 (2.3) | 10 (2.3) |  |
| Insurance Coverage, N (%) |  |  | 0.0001 |
| Commercial insurance | 187 (54.8) | 266 (59.9) |  |
| Medicaid | 20 (5.9) | 56 (12.6) |  |
| Medicare | 134 (39.3) | 122 (27.5) |  |
| Smoking, N (%) |  |  | 0.37 |
| Current | 27 (7.9) | 25 (5.6) |  |
| Former | 100 (29.3) | 125 (28.2) |  |
| Never | 214 (62.8) | 294 (66.2) |  |
| Specialty status, N (%) |  |  | 0.0057 |
| Endocrinology | 104 (30.5) | 179 (40.3) |  |
| Other | 237 (69.5) | 265 (59.7) |  |
| Last encounter type, N (%) |  |  | 0.98 |
| Hospital Encounter | 32 (9.4) | 40 (9.0) |  |
| Office Visit | 249 (73.0) | 327 (73.6) |  |
| Telemedicine | 60 (17.6) | 77 (17.3) |  |
| Last visit year, N (%) |  |  | 0.52 |
| 2015 | 20 (5.9) | 18 (4.1) |  |
| 2016 | 30 (8.8) | 30 (6.8) |  |
| 2017 | 42 (12.3) | 53 (11.9) |  |
| 2018 | 47 (13.8) | 72 (16.2) |  |
| 2019 | 52 (15.2) | 81 (18.2) |  |
| 2020 | 150 (44.0) | 190 (42.8) |  |

^a^ HbA1c > 8.5% in the initial year and ≤ 8.5% in subsequent 3 years

^b^ HbA1c > 8.5% in all four studied years
